# Research prioritization of men’s health and urologic diseases

**DOI:** 10.1590/S1677-5538.IBJU.2016.0047

**Published:** 2017

**Authors:** Tyler Okland, Chante Karimkhani, Hannah Pederson, Lindsay N Boyers, Mark D. Sawyer, Kyle O. Rove, McCabe C. Kenny, Steven Steinberg, Mohsen Naghavi, Robert P. Dellavalle

**Affiliations:** 1 University of Colorado School of Medicine, Aurora, Colorado, USA;; 2 College of Physicians and Surgeons, Columbia University, New York, NY, USA;; 3 School of Medicine, Georgetown University, Washington, District of Columbia, USA;; 4 Urology Service, Unites States Department of Veterans Affairs, Eastern Colorado Health Care System, Denver, Colorado, USA;; 5 Department of Urology, University of Colorado Anschutz Medical Campus, Aurora, Colorado, USA;; 6 Institute for Health Metrics and Evaluation, University of Washington, Seattle, Washington, USA;; 7 Department of Dermatology, University of Colorado Anschutz Medical Campus, Aurora, Colorado, USA;; 8 Dermatology Service, Unites States Department of Veterans, Eastern Colorado Health Care System, Denver, Colorado, USA;; 9 Department of Epidemiology, Colorado School of Public Health, University of Colorado Anschutz Medical Campus, Aurora, Colorado, USA

**Keywords:** Men’s Health, Urologic Diseases, Neoplasms, Infertility, Male

## Abstract

**Objectives:**

We sought to determine whether disease representation in the Cochrane Database of Systematic Reviews (CDSR) reflects disease burden, measured by the Global Burden of Disease (GBD) Study as disability-adjusted life-years (DALYs).

**Materials and Methods:**

Two investigators performed independent assessment of ten men’s health and urologic diseases (MHUDs) in CDSR for systematic review and protocol representation, which were compared with percentage of total 2010 DALYs for the ten conditions. Data were analyzed for correlation using Spearman rank analysis.

**Results:**

Nine of ten MHUDs were represented by at least one CDSR review. There was a poor and statistically insignificant positive correlation between CDSR representation and disease burden (rho = 0.42, p = 0.23). CDSR representation was aligned with disease burden for three conditions, greater than disease burden for one condition, and less than disease burden for six conditions.

**Conclusions:**

These results yield high-quality estimates to inform future research prioritization for MHUDs. While prioritization processes are complex and multi-faceted, disease burden should be strongly considered. Awareness of research priority setting has the potential to minimize research disparities on a global scale.

## INTRODUCTION

**Figure 1:**
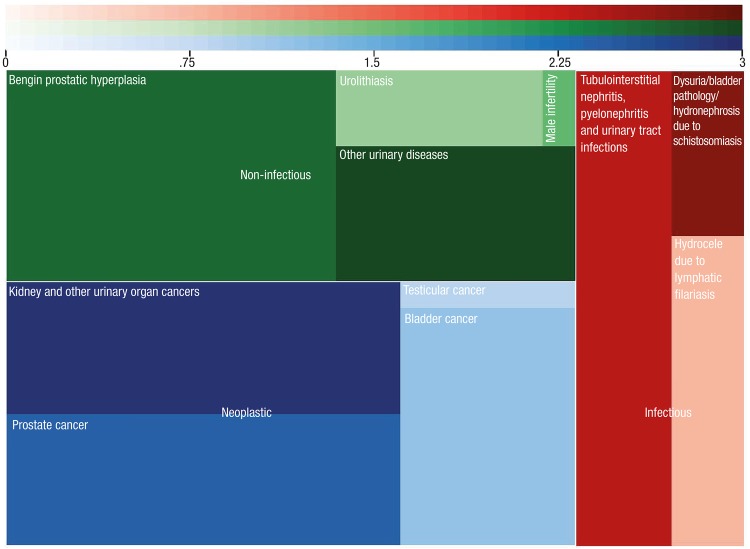
Square pie chart representing percent of total DALY for ten men’s health and urologic diseases; area of each square/rectangle represents percentage of total burden.

In order to achieve effective clinical research, scarce research funds must be distributed to appropriate diseases in order to maximize health benefits to the represented population. Systematic approaches to inform research prioritization include identifying and prioritizing research questions, recognizing existing research, and setting goals for primary research (1, 2). A derivative of this approach is to value major diseases, injuries, and risk factors based on their burden to society (3). Spearheaded by the Institute for Health Metrics and Evaluation (IHME), the Global Burden of Disease (GBD) 2010 Study estimates the burden of 291 diseases and injuries across 187 countries from 1990 to 2010 (4, 5). The metric of disability-adjusted life years (DALYs), in which 1 DALY is equivalent to 1 year of healthy life lost, allows for descriptive global epidemiology of a wide array of disease states. The following ten men’s health and urologic diseases (MHUDs) were studied by GBD on the basis of prevalence, common case definitions, and data availability: tubulointerstitial nephritis, pyelonephritis, and urinary tract infections; kidney and other urinary organ cancers; urolithiasis; male infertility; benign prostatic hyperplasia; prostate cancer; testicular cancer; hydrocele due to lymphatic filariasis; dysuria/bladder pathology/hydronephrosis due to schistosomiasis; and bladder cancer (Figure-1).

**green =** non-infectious conditions, **red =** infectious conditions, and **blue =** neoplastic conditions

Systematic reviews are the cornerstone of evidence-based medicine, yet few efforts have been made to assess whether the prioritization of systematic reviews reflect global disease burden (6). The Cochrane Database of Systematic Reviews (CDSR) produces systematic reviews and protocols (published proposals for future systematic reviews) across all medical specialties as well as health systems, public health, and child development. Cochrane systematic reviews undergo exhaustive editorial processing, are more methodologically rigorous, and are updated more frequently than non-Cochrane reviews and paper-based journals (7, 8). Prior studies have evaluated the association between broad categories of disease burden with randomized trials and Cochrane systematic reviews (9-12). This study will assess whether the CDSR representation of ten MHUDs corresponds to GBD 2010 disability estimates.

## MATERIALS AND METHODS

ICD-10 code definitions for the ten MHUDs have been previously published and were used to generate search terms, which were entered into the “title, abstract, keywords” CDSR search function (5, 13). Systematic reviews and protocols were considered to determine MHUD representation in CDSR, according to abstract subject content. Online publication date, the number of studies included in each systematic review, and the particular Cochrane review group that published the review or protocol were collected.

Two authors (T.O and H.P.) collected data independently during February 2015. DALY metrics for each of the ten MHUDs, expressed as percentages of total DALY’s of all 291 conditions measured in GBD 2010, were obtained from the GBD Compare interactive time plot, available at <http://viz.healthmetricsandevaluation.org/gbd-compare/>. Spearman rank correlation analysis was performed to assess statistical dependence between CDSR representation and disease burden. Rho, a coefficient ranging from-1 (strong negative correlation) to +1 (strong positive correlation), is interpreted with a two-tailed p-value. A line-of-best-fit was also generated between CDSR representation and % of total DALYs.

As this study did not involve human subjects, institutional review board approval was not necessary.

## RESULTS

Nine of the ten MHUD conditions studied in GBD 2010 were represented by at least one systematic review. A total of 116 systematic reviews and protocols published by nine Cochrane review groups represented the ten MHUDs (Supporting Tables 1 and 2 for included and excluded titles, respectively). The majority of reviews and protocols covered tubulointerstitial nephritis, pyelonephritis, and urinary tract infections (n=46). Hydrocele due to lymphatic filariasis had no representation in CDSR. Of the ten MHUDs, benign prostatic hyperplasia had the greatest global disease burden (0.2%) while male infertility had the lowest (0.007%) Table-1.

**Table 1:** Men’s health and urologic diseases studied by GBD 2010 with corresponding ICD-10 codes, search terms, number of systematic reviews (R) and protocols (P) in CDSR, percent of total DALYs (arranged in order of decreasing % of total DALY), and number of studies included in Cochrane reviews.

Condition	ICD-10 code	Search terms	Number of cochrane reviews (R) & protocols (P)	% total 210 dalys (out of 291 conditions)	Number of studies in cochrane review
Benign prostatic hyperplasia	N40	“benign prostatic hyperplasia” “median bar” “prostatic hyperplasia” “adenofibromatous hypertrophy of prostrate” “hypertrophy of prostate “prostatic obstruction”	9 (7 R, 2 P)	0.2%	35
Prostate cancer	C61, D07.5, D40.0	“prostate cancer” “prostatic carcinoma in situ” “prostate neoplasm”	23 (22 R, 1 P)	0.15%	411
Kidney and other urinary organ cancers	C64-C66, D41.0-D41.2	“kidney cancer” “neoplasm of the ureter” “neoplasm of the kidney” “neoplasm of the renal pelvis”	4 (4 R)	0.15%	63
Tubulointerstitial nephritis, pyelonephritis, and urinary tract infections	N10-N12, N15.1-N15.9, N30, N34, N39.0	“tubulointerstitial nephritis” “pyelonephritis” “urinary tract infections” “infectious interstitial nephritis” “pyelitis” “balkan nephropathy” “renal and perinephric abscess” “cystitis” “trigonitis” “urethral abscess” “urethritis”	46 (41 R, 5 P)	0.13%	529
Bladder cancer	C67, D09.0*, D41.4	“bladder cancer” “bladder carcinoma” “bladder neoplasm”	11 (10 R, 1 P)	0.12%	106
Hydrocele due to lymphatic filariasis	B74 (except B74.3, B74.4, B74.8, B74.9)	“lymphatic filariasis” [hydrocele]	0	0.064%	0
Urolithiasis	N20-N23	“urolithiasis” “urinary stones” “nephrolithiasis” “kidney stones” “ureterolithiasis” “cystolithiasis” “bladder stones”	10 (8 R, 2 P)	0.045%	59
Dysuria/bladder pathology/hydronephrosis due to schistosomiasis	B65	“schistosomiasis” [dysuria, bladder, hydronephrosis]	4 (2 R, 2 P)	0.034%	35
Testicular cancer	C62, D40.1	“testicular cancer” “malignant neoplasm of testis”	1 R	0.013%	0
Male infertility	N46	“azoospermia” “oligospermia”	8 R	0.007	95

**Table:** Supporting Table 1

Injury or Trauma	Review (R) or Protocol (P) Title	Cochrane Group	Number of Studies	Year of Online Publication
Tubulo-interstitial nephritis, pyelonephritis, and urinary tract infections	Antibiotics for acute pyelonephritis in children (R)	Renal Group	27	2007
	Antibiotics for asymptomatic bacteriuria in pregnancy (R)	Pregnancy and Childbirth Group	14	2007
	Treatments for symptomatic urinary tract infections during pregnancy (R)	Pregnancy and Childbirth Group	10	2011
	Duration of treatment for asymptomatic bacteriuria during pregnancy (R)	Pregnancy and Childbirth Group	13	2011
	Routine blood cultures in the management of pyelonephritis in pregnancy for improving outcomes (R)	Renal Group	0	2015
	Procalcitonin , C-reactive protein , and erythrocyte sedimentation rate for the diagnosis of acute pyelonephritis in children (R)	Renal Group	17	2015
	Duration of antibacterial treatment for uncomplicated urinary tract infection in women (R)	Renal Group	32	2005
	Interventions for preventing recurrent urinary tract infection during pregnancy (R)	Pregnancy and Childbirth Group	1	2012
	Long-term antibiotics for preventing recurrent urinary tract infection in children (R)	Renal Group	12	2011
	Treatments for symptomatic urinary tract infections during pregnancy (R)	Pregnancy and Childbirth Group	10	2011
	Prophylactic antibiotics to reduce the risk of urinary tract infections after urodynamic studies (R)	Incontinence Group	9	2012
	Types of indwelling urethral catheters for short-term catheterization in hospitalized adults (R)	Incontinence Group	26	2014
	Antibiotics for treating lower urinary tract infection in children (R)	Renal Group	16	2012
	Methenamine hippurate for preventing urinary tract infections (R)	Renal Group	13	2012
	Estrogens for preventing recurrent urinary tract infection in postmenopausal women (R)	Renal Group	9	2008
	Short versus standard duration oral antibiotic therapy for acute urinary tract infection in children (R)	Renal Group	10	2003
	Urinary catheter policies for long-term bladder drainage (R)	Incontinence Group	8	2012
	Antimicrobial agents for treating uncomplicated urinary tract infection in women (R)	Renal Group	21	2010
	Modes of administration of antibiotics for symptomatic severe urinary tract infections (R)	Renal Group	15	2007
	Antibiotic duration for treating uncomplicated , symptomatic lower urinary tract infections in elderly women (R)	Renal Group	15	2008
	Antibiotic prophylaxis for short-term catheter bladder drainage in adults (R)	Incontinence Group	6	2013
	Cranberries for treating urinary tract infections (R)	Renal Group	0	1998
	Antibiotics for preventing recurrent urinary tract infection in non-pregnant women (R)	Renal Group	19	2004
	Cranberries for preventing urinary tract infections (R)	Renal Group	24	2012
	Routine neonatal circumcision for the prevention of urinary tract infections in infancy (R)	Neonatal Group	0	2012
	Urinary catheter policies for short-term bladder drainage in adults (R)	Incontinence Group	17	2005
	Intermittent catheterization for long-term bladder management (R)	Incontinence Group	31	2014
	Antibiotic prophylaxis for transrectal prostate biopsy (R)	Prostatic Diseases and Urologic Cancers Group	19	2011
	Short term urinary catheter policies following urogenital surgery in adults (R)	Incontinence Group	39	2006
	Washout policies in long-term indwelling urinary catheterization in adults (R)	Incontinence Group	5	2010
	Routine intraoperative ureteric stenting for kidney transplant recipients (R)	Renal Group	7	2013
	Interventions for primary vesicoureteric reflux (R)	Renal Group	20	2011
	Types of indwelling urinary catheters for long-term bladder drainage in adults (R)	Incontinence Group	3	2012
	Laser prostatectomy for benign prostatic obstruction (R)	Prostatic Diseases and Urologic Cancers Group	20	2000
	Quinolones for uncomplicated acute cystitis in women (R)	Renal Group	11	2006
	Drugs for treatment of urinary retention after surgery in adults (R)	Incontinence Group	7	2010
	Indwelling bladder catheterization as part of intraoperative and postoperative care for caesarean section (R)	Pregnancy and Childbirth Group	5	2014
	Dietary interventions for preventing complications in idiopathic hypercalciuria (R)	Renal Group	5	2014
	Interventions for covert bacteriuria in children (R)	Renal Group	3	2012
	Pharmacological interventions for preventing complications in idiopathic hypercalciuria (R)	Renal Group	5	2009
	Different antibiotic regimens for treating asymptomatic bacteriuria in pregnancy (R)	Pregnancy and Childbirth Group	5	2010
	Urinary alkalization for uncomplicated urinary tract infection (P)	Renal Group	N/A	2013
	Dimercaptosuccinic acid scan versus ultrasound in screening for vesicoureteral reflux among children with urinary tract infections (P)	Renal Group	N/A	2013
	Chinese herbal medicine for treating recurrent urinary tract infections in women (P)	Renal Group	N/A	2013
	Probiotics for preventing urinary tract infection in people with neuropathic bladder (P)	Renal Group	N/A	2013
	Probiotics for preventing urinary tract infections in adults and children (P)	Renal Group	N/A	2010
Kidney and other urinary organ cancers	Targeted therapy for advanced renal cell carcinoma (R)	Prostatic Diseases and Urologic Cancers Group	25	2008
	Immunotherapy for advanced renal cell cancer (R)	Prostatic Diseases and Urologic Cancers Group	37	2004
	Surgical management of localized renal cell carcinoma (R)	Prostatic Diseases and Urologic Cancers Group	0	2010
	Surgical management for upper urinary tract transitional cell carcinoma (R)	Prostatic Diseases and Urologic Cancers Group	1	2011
Urolithiasis	Extracorporeal shock wave lithotripsy (ESWL ) versus percutaneous nephrolithotomy (PCNL ) or retrograde intrarenal surgery (RIRS ) for kidney stones (R)	Renal Group	5	2014
	Dietary interventions for preventing complications in idiopathic hypercalciuria (R)	Renal Group	5	2014
	Extracorporeal shock wave lithotripsy (ESWL ) versus ureteroscopic management for ureteric calculi (R)	Renal Group	7	2012
	Pharmacological interventions for preventing complications in idiopathic hypercalciuria (R)	Renal Group	5	2009
	Fluids and diuretics for acute ureteric colic (R)	Renal Group	2	2012
	Water for preventing urinary stones (R)	Renal Group	1	2012
	Alpha-blockers as medical expulsive therapy for ureteral stones (R)	Renal Group	32	2014
	Percussion , diuresis , and inversion therapy for the passage of lower pole kidney stones following shock wave lithotripsy (R)	Renal Group	2	2013
	Analgesia for patients undergoing shockwave lithotripsy for urinary stones (P)	Renal Group	N/A	2012
	Interventions for treating urinary stones in children (P)	Renal Group	N/A	2013
Male infertility	Intra-uterine insemination for male subfertility (R)	Menstrual Disorders and Subfertility Group	8	2007
	Antioxidants for male subfertility (R)	Menstrual Disorders and Subfertility Group	48	2014
	Surgery or embolization for varicoceles in subfertile men (R)	Menstrual Disorders and Subfertility Group	10	2012
	Regular (ICSI ) versus ultra-high magnification (IMSI ) sperm selection for assisted reproduction (R)	Menstrual Disorders and Subfertility Group	9	2013
	Intra-uterine insemination for unexplained subfertility (R)	Menstrual Disorders and Subfertility Group	8	2012
	Techniques for surgical retrieval of sperm prior to intra-cytoplasmic sperm injection (ICSI ) for azoospermia (R)	Menstrual Disorders and Subfertility Group	2	2008
	Cervical insemination versus intra-uterine insemination of donor sperm for subfertility (R)	Menstrual Disorders and Subfertility Group	4	2008
	Gonadotrophins for idiopathic male factor subfertility (R)	Menstrual Disorders and Subfertility Group	6	2013
Benign prostatic hyperplasia	Pygeum africanum for benign prostatic hyperplasia (R)	Prostatic Diseases and Urologic Cancers Group	18	1998
	Finasteride for benign prostatic hyperplasia (R)	Prostatic Diseases and Urologic Cancers Group	23	2010
	Beta-sitosterols for benign prostatic hyperplasia (R)	Prostatic Diseases and Urologic Cancers Group	4	1999
	Serenoa repens for benign prostatic hyperplasia (R)	Prostatic Diseases and Urologic Cancers Group	32	2012
	Naftopidil for the treatment of lower urinary tract symptoms compatible with benign prostatic hyperplasia (R)	Prostatic Diseases and Urologic Cancers Group		
	Microwave thermotherapy for benign prostatic hyperplasia (R)	Prostatic Diseases and Urologic Cancers Group	15	2012
	Phosphodiesterase inhibitors for lower urinary tract symptoms consistent with benign prostatic hyperplasia (P)	Prostatic Diseases and Urologic Cancers Group	N/A	2012
	Laser prostatectomy for benign prostatic obstruction (R)	Prostatic Diseases and Urologic Cancers Group	20	2000
	Bipolar versus monopolar transurethral resection of the prostate for lower urinary tract symptoms secondary to benign prostatic obstruction (P)	Prostatic Diseases and Urologic Cancers Group	N/A	2014
Prostate cancer	5-alpha-reductase inhibitors for prostate cancer prevention (R)	Prostatic Diseases and Urologic Cancers Group	9	2008
	Screening for prostate cancer (R)	Prostatic Diseases and Urologic Cancers Group	5	2013
	Lycopene for the prevention of prostate cancer (R)	Prostatic Diseases and Urologic Cancers Group	3	2011
	Psychosocial interventions for men with prostate cancer (R)	Prostatic Diseases and Urologic Cancers Group	19	2013
	Radical prostatectomy versus watchful waiting for prostate cancer (R)	Prostatic Diseases and Urologic Cancers Group	2	2010
	Bisphosphonates for advanced prostate cancer (R)	Prostatic Diseases and Urologic Cancers Group	10	2006
	Cryotherapy for localized prostate cancer (R)	Prostatic Diseases and Urologic Cancers Group	8	2007
	Neo- adjuvant and adjuvant hormone therapy for localized and locally advanced prostate cancer (R)	Prostatic Diseases and Urologic Cancers Group	21	2006
	Early versus deferred androgen suppression in the treatment of advanced prostatic cancer (R)	Prostatic Diseases and Urologic Cancers Group	4	2001
	Chemotherapy for hormone-refractory prostate cancer (R)	Prostatic Diseases and Urologic Cancers Group	47	2006
	Maximal androgen blockade for advanced prostate cancer (R)	Prostatic Diseases and Urologic Cancers Group	20	1999
	Adjuvant radiotherapy following radical prostatectomy for prostate cancer (R)	Prostatic Diseases and Urologic Cancers Group	3	2011
	Intermittent versus continuous androgen suppression for prostatic cancer (R)	Prostatic Diseases and Urologic Cancers Group	5	2007
	Low-dose rate brachytherapy for men with localized prostate cancer (R)	Prostatic Diseases and Urologic Cancers Group	1	2011
	Interventions for sexual dysfunction following treatments for cancer (R)	Pain, Palliative and Supportive Care Group	11	2007
	Selenium for preventing cancer (R)	Gynaecological Cancer Group	55	2014
	Green tea (Camellia sinensis) for the prevention of cancer (R)	Gynaecological Cancer Group	51	2009
	Exercise for the management of cancer-related fatigue in adults (R)	Pain, Palliative and Supportive Care Group	56	2012
	Laparoscopic versus open prostatectomy for the treatment of localized prostate cancer (P)	Prostatic Diseases and Urologic Cancers Group	N/A	2012
	Non-steroidal antiandrogen monotherapy compared with luteinizing hormone –releasing hormone agonists or surgical castration monotherapy for advanced prostate cancer (R)	Prostatic Diseases and Urologic Cancers Group	11	2014
	Conservative management for postprostatectomy urinary incontinence (R)	Incontinence Group	50	2015
	Surgery for stress urinary incontinence due to presumed sphincter deficiency after prostate surgery (R)	Incontinence Group	1	2014
	Antibiotic prophylaxis for transrectal prostate biopsy (R)	Prostatic Diseases and Urologic Cancers Group	19	2011
Testicular cancer	Screening for testicular cancer (R)	Prostatic Diseases and Urologic Cancers Group	0	2011
Bladder cancer	Intravesical gemcitabine for non-muscle invasive bladder cancer (R)	Prostatic Diseases and Urologic Cancers Group	6	2012
	Intravesical Bacillus Calmette-Guérin versus epirubicin for Ta and T1 bladder cancer (R)	Prostatic Diseases and Urologic Cancers Group	5	2011
	Gemcitabine for unresectable , locally advanced or metastatic bladder cancer (R)	Prostatic Diseases and Urologic Cancers Group	6	2011
	Intravesical Bacillus Calmette-Guérin in Ta and T1 bladder cancer (R)	Prostatic Diseases and Urologic Cancers Group	6	2000
	Neo-adjuvant chemotherapy for invasive bladder cancer (R)	Prostatic Diseases and Urologic Cancers Group	11	2004
	Intravesical Bacillus Calmette-Guérin versus mitomycin C for Ta and T1 bladder cancer (R)	Prostatic Diseases and Urologic Cancers Group	7	2003
	Surgery versus radiotherapy for muscle invasive bladder cancer (R)	Prostatic Diseases and Urologic Cancers Group	3	2001
	Adjuvant chemotherapy for invasive bladder cancer (individual patient data) (R)	Prostatic Diseases and Urologic Cancers Group	6	2006
	Green tea (Camellia sinensis) for the prevention of cancer (R)	Gyanecological Cancer Group	51	2009
	Perioperative nutrition for the treatment of bladder cancer by radical cystectomy (P)	Prostatic Diseases and Urologic Cancers Group	N/A	2012
	Urinary diversion and bladder reconstruction /replacement using intestinal segments for intractable incontinence or following cystectomy (R)	Incontinence Group	5	2012
Hydrocele due to lymphatic filariasis				
Dysuria/bladder pathology/hydronephrosis due to schistosomiasis	Drugs for treating urinary **schistosomiasis** (R)	Infectious Diseases Group	30	2014
	Therapeutic and prophylactic drug interventions for **Schistosomiasis** japonicum (P)	Infectious Diseases Group	N/A	2012
	Rapid screening and diagnostic tests for human **schistosomiasis** in endemic areas (P)	Infectious Diseases Group	N/A	2012
	Urinary diversion and bladder reconstruction/replacement using intestinal segments for intractable incontinence or following cystectomy (R)	Incontinence Group	5	2012

**Table:** Supporting Table 2:

Condition	Review (R) or Protocol (P)
Tubulo-interstitial nephritis, pyelonephritis, and urinary tract infections	Interventions to improve professional adherence to guidelines for prevention of device-related infections (R)
	Mechanical dilatation of the cervix at non-labour caesarean section for reducing postoperative morbidity (R)
	Antibiotic prophylaxis versus no prophylaxis for preventing infection after cesarean section (R)
	Traditional suburethral sling operations for urinary incontinence in women (R)
	Antibiotic prophylaxis for surgery for proximal femoral and other closed long bone fractures (R)
	Mupirocin ointment for preventing Staphylococcus aureus infections in nasal carriers (R)
	Rituximab for relapsing-remitting multiple sclerosis (R)
	Beta lactam antibiotic monotherapy versus beta lactam -aminoglycoside antibiotic combination therapy for sepsis (R)
	Habit retraining for the management of urinary incontinence in adults (R)
	Preoperative skin antiseptics for preventing surgical wound infections after clean surgery (R)
	Antibiotic prophylaxis for cirrhotic patients with upper gastrointestinal bleeding (R)
	Antimicrobial therapy for chronic bacterial prostatitis (R)
	Cyanoacrylate microbial sealants for skin preparation prior to surgery (R)
	Perioperative increase in global blood flow to explicit defined goals and outcomes following surgery (R)
	Interventions to improve antibiotic prescribing practices in ambulatory care (R)
	Teriflunomide for multiple sclerosis (R)
	Laquinimod for multiple sclerosis (R)
	Mitoxantrone for multiple sclerosis (R)
	Efficacy and safety of cesarean delivery for prevention of mother-to-child transmission of HIV-1 (R)
	Surgical approach to hysterectomy for benign gynecological disease (R)
	Interventions for preventing mastitis after childbirth (R)
	Laparoscopy versus laparotomy for benign ovarian tumor (R)
	Valproate preparations for agitation in dementia (R)
	Regional versus general anesthesia for caesarean section (R)
	Interventions for promoting the initiation of breastfeeding (R)
	Protocolized versus non- protocolized weaning for reducing the duration of mechanical ventilation in critically ill adult patients (R)
	In-hospital care pathways for stroke (R)
	Schedules for home visits in the early postpartum period (R)
	Short term benefits for laparoscopic colorectal resection (R)
	Diaphragm versus diaphragm with spermicides for contraception (R)
	Cervical cap versus diaphragm for contraception (R)
Kidney and other urinary organ cancers	Early and late renal adverse effects after potentially nephrotoxic treatment for childhood cancer (R)
	Urate oxidase for the prevention and treatment of tumor lysis syndrome in children with cancer (R)
	Bisphosphonates and other bone agents for breast cancer (R)
	Interventions for preventing non-melanoma skin cancers in high-risk groups (R)
	Perioperative blood transfusions and recurrence of colorectal cancer (R)
	Medical interventions for the prevention of platinum-induced hearing loss in children with cancer (R)
	Cisplatin versus carboplatin in combination with third-generation drugs for advanced non-small cell lung cancer (R)
	Immunosuppressive T-cell antibody induction for heart transplant recipients (R)
	Concomitant hydroxyurea plus radiotherapy versus radiotherapy for carcinoma of the uterine cervix (R)
	Amphotericin B versus fluconazole for controlling fungal infections in neutropenic cancer patients (R)
	HMG CoA reductase inhibitors (statins) for dialysis patients (R)
	Urinary diversion and bladder reconstruction /replacement using intestinal segments for intractable incontinence or following cystectomy (R)
	Pharmacological interventions for pruritus in adult palliative care patients (R)
	Homocysteine-lowering interventions for preventing cardiovascular events (R)
	HMG CoA reductase inhibitors (statins ) for kidney transplant recipients (R)
	Vitamin D supplementation for prevention of mortality in adults (R)
	Vitamin D and vitamin D analogues for preventing fractures in post-menopausal women and older men (R)
	Antibody induction therapy for lung transplant recipients (R)
	First-line tandem high-dose chemotherapy and autologous stem cell transplantation versus single high-dose chemotherapy and autologous stem cell transplantation in multiple myeloma , a systematic review of controlled studies (R)
	Antibiotic prophylaxis for preventing post solid organ transplant tuberculosis (R)
	Tacrolimus versus cyclosporin as primary immunosuppression for lung transplant recipients (R)
	Bisphosphonates in multiple myeloma: a network meta-analysis (R)
	Darbepoetin for the anemia of chronic kidney disease (R)
	Selenium for preventing cancer (R)
	Hyperbaric oxygenation for tumor sensitization to radiotherapy (R)
	Adjuvant radiotherapy following radical prostatectomy for prostate cancer (R)
	Exenterative surgery for recurrent gynecological malignancies (R)
	Laparoscopically assisted radical vaginal hysterectomy versus radical abdominal hysterectomy for the treatment of early cervical cancer (R)
	Laparoscopy versus laparotomy for the management of early stage endometrial cancer (R)
	Laparoscopic versus open total mesorectal excision for rectal cancer (R)
	Adjuvant radiotherapy for stage I endometrial cancer (R)
	Cryotherapy for localized prostate cancer (R)
	Spinal cord stimulation for cancer -related pain in adults (R)
	Chemoradiation for advanced primary vulval cancer (R)
	High dose rate versus low dose rate intracavity brachytherapy for locally advanced uterine cervix cancer (R)
	Surgical management for upper urinary tract transitional cell carcinoma (R)
	Cholecystectomy for patients with silent gallstones (R)
	Drugs for treating urinary schistosomiasis (R)
	Non-surgical interventions for late radiation cystitis in patients who have received radical radiotherapy to the pelvis (R)
	Cranberries for preventing urinary tract infections (R)
	Radioiodine therapy for differentiated thyroid carcinoma with thyroglobulin positive and radioactive iodine negative metastases (R)
	Conservative management for postprostatectomy urinary incontinence (R)
	Surgery for stress urinary incontinence due to presumed sphincter deficiency after prostate surgery (R)
	The role of alpha blockers prior to removal of urethral catheter for acute urinary retention in men (R)
Urolithiasis	Pegloticase for chronic gout (R)
	Fluids and diuretics for acute ureteric colic (R)
	Vitamin D supplementation for prevention of mortality in adults (R)
	Chinese medicinal herbs for cholelithiasis (R)
	Thyroid hormones for acute kidney injury (R)
	Vitamin D and vitamin D analogues for preventing fractures in post-menopausal women and older men (R)
Male infertility	Intra-cytoplasmic sperm injection versus conventional techniques for oocyte insemination during in vitro fertilisation in couples with non-male subfertility (R)
	Oral 5-aminosalicylic acid for induction of remission in ulcerative colitis (R)
	Oral 5-aminosalicylic acid for maintenance of remission in ulcerative colitis (R)
	Intrauterine insemination versus fallopian tube sperm perfusion for non-tubal infertility (R)
	Vasectomy occlusion techniques for male sterilization (R)
	Steroid hormones for contraception in men (R)
Benign prostatic hyperplasia	Interventions for chronic abacterial prostatitis (R)
Prostate cancer	Interventions for improving the adoption of shared decision making by healthcare professionals (R)
Testicular cancer	Chemotherapy for malignant germ cell ovarian cancer in adult patients with early stage, advanced and recurrent disease (R)
Bladder cancer	Cholecystectomy for patients with silent gallstones (R)
	Hyperbaric oxygenation for tumor sensitization to radiotherapy (R)
	Drugs for treating urinary schistosomiasis (R)
	High dose rate versus low dose rate intracavity brachytherapy for locally advanced uterine cervix cancer (R)
	Adjuvant radiotherapy for stage I endometrial cancer (R)
	Laparoscopy versus laparotomy for the management of early stage endometrial cancer (R)
	Selenium for preventing cancer (R)
	Radioiodine therapy for differentiated thyroid carcinoma with thyroglobulin positive and radioactive iodine negative metastases (R)
	Chemoradiation for advanced primary vulval cancer (R)
	Laparoscopic versus open total mesorectal excision for rectal cancer (R)
	Spinal cord stimulation for cancer-related pain in adults (R)
Hydrocele due to lymphatic filariasis	Albendazole for lymphatic filariasis (R)
	Diethylcarbamazine (DEC)-medicated salt for community-based control of lymphatic filariasis (R)
Dysuria/bladder pathology/hydronephrosis due to schistosomiasis	Metrifonate for Alzheimer's disease (R)
	Drugs for treating Schistosoma mansoni infection (R)

Reviews and protocols representing the ten MHUDs were published by the following Cochrane review groups: Prostatic Diseases and Urologic Cancers Group (n=43); Renal Group (36); Incontinence Group (14); Menstrual Disorders and Subfertility Group (8); Pregnancy and Childbirth Group (7); Infectious Diseases Group (3); Pain, Palliative and Supportive Care Group (2); Gynecological Cancer Group (2); and Neonatal Group (1).

**Figure 2:**
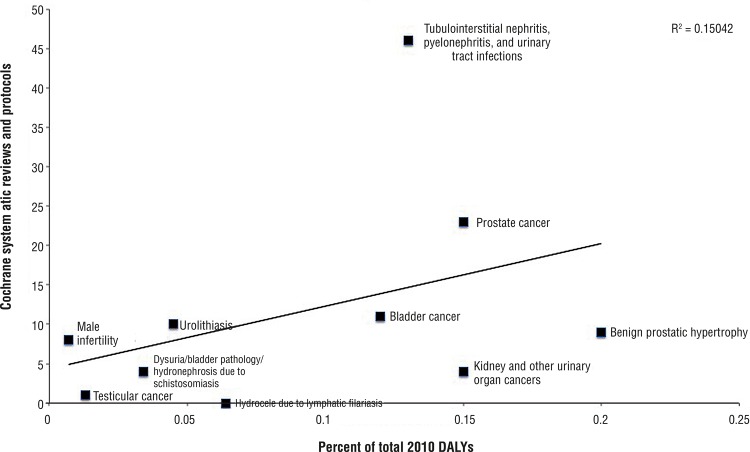
Comparison of men’s health and urologic disease representation in the Cochrane Database of Systematic Reviews with percent of 2010 DALYs from 291 conditions studied by GBD 2010.

Spearman rank correlation testing between CDSR representation and DALY metrics revealed poor positive correlation that was statistically insignificant (rho=0.41, p=0.21). The majority of the MHUDs (6) were under-represented in CDSR as compared to GBD DALY (Figure-2). Most of the systematic reviews and protocols (58.6%) were published from 2011 to 2015 while 37.9% were published from 2000 to 2010; only 4 reviews were published prior to 2000. Maintaining systematic reviews up-to-date is critical to deliver consensus statements on current world literature that ultimately impact clinical decisions and patient outcomes.

Representation of tubulointerstitial nephritis, pyelonephritis, and urinary tract infections exceeded GBD disease burden. This disease category also had the greatest number of cumulative studies informing its systematic reviews (529). The one systematic review representing testicular cancer, entitled “Screening for testicular cancer,” found no randomized controlled trials in the literature. Systematic reviews that find no suitable trials to address their objectives uncover areas for much-needed, high-quality research.

The World Health Organization (WHO) classifies two of the MHUDs as neglected tropical diseases: dysuria/bladder pathology/hydronephrosis due to schistosomiasis and hydrocele due to lymphatic filariasis (14). It is important to note that just as the DALY metrics reported for these two diseases include only burden due to the MHUD morbidity (dysuria, bladder pathology, hydronephrosis, hydrocele), systematic reviews were only considered representative if they included assessment of the MHUD pathology.

## DISCUSSION

We acknowledge several limitations of our study. The scope of CDSR systematic reviews is subject to variability. For instance, authors may prepare one large review of multiple interventions (lumping) or several reviews of individual interventions (splitting). Therefore, treating a systematic review or protocol as one measurement unit may not be entirely accurate for every topic. While beyond the scope of this limited study, further exploration is warranted into potential underrepresentation of certain conditions.

There remains a lack of transparency in publications and databases on the quality of data and criteria involved in prioritization decisions (15). Other important factors in priority setting include availability of research funds, knowledge gap, and impact on disadvantaged populations. Research prioritization is also inherently political and dependent on financial backing, which further demonstrates the importance of a transparent process. Attention and awareness of priority setting has the potential to minimize research disparities and, ultimately, impact populations at a global scale.
